# Emotional and informational social support from health visitors and breastfeeding outcomes in the UK

**DOI:** 10.1186/s13006-023-00551-7

**Published:** 2023-03-07

**Authors:** A Chambers, EH Emmott, S Myers, AE Page

**Affiliations:** 1 Department of Population Health, London School of Hygiene and Tropical Medicine, London, UK; 2 UCL Anthropology, University College London, London, UK; 3 BirthRites Independent Max Planck Research Group, Max Planck Institute for Evolutionary Anthropology, Leipzig, Germany

**Keywords:** Infant feeding, Social support, Breast/chest-feeding, Health visitors, UK

## Abstract

**Background:**

Shorter breastfeeding duration is associated with detrimental consequences for infant health/development and maternal health. Previous studies suggest social support is essential in maintaining breast/chest-feeding and helping to improve general infant feeding experiences. Public health bodies therefore work to support breastfeeding in the UK, yet UK breastfeeding rates continue to be one of the lowest globally. With this, a better understanding of the effectiveness and quality of infant feeding support is required. In the UK, health visitors (community public health nurses specialising in working with families with a child aged 0–5 years) have been positioned as one of the key providers of breast/chest-feeding support. Research evidence suggests that both inadequate informational support and poor/negative emotional support can lead to poor breastfeeding experiences and early breastfeeding cessation. Thus, this study tests the hypothesis that emotional support from health visitors moderates the relationship between informational support and breastfeeding duration/infant feeding experience among UK mothers.

**Methods:**

We ran cox and binary logistic regression models on data from 565 UK mothers, collected as part of a 2017–2018 retrospective online survey on social support and infant feeding.

**Results:**

Informational support, compared to emotional support, was a less important predictor of both breastfeeding duration and experience. Supportive emotional support with unhelpful or absent informational support was associated with the lowest hazard of breastfeeding cessation before 3 months. Results for breastfeeding experience followed similar trends, where positive experience was associated with *supportive* emotional and *unhelpful* informational support. Negative experiences were less consistent; however, a higher probability of negative experience was found when both types of support were reported as unsupportive.

**Conclusions:**

Our findings point to the importance of health visitors providing emotional support to bolster the continuation of breastfeeding and encourage a positive subjective experience of infant feeding. The emphasis of emotional support in our results encourages increased allocation of resources and training opportunities to ensure health visitors are able to provide enhanced emotional support. Lowering health visitors caseloads to allow for personalised care is just one actionable example that may improve breastfeeding outcomes in the UK.

**Supplementary Information:**

The online version contains supplementary material available at 10.1186/s13006-023-00551-7.

## Background

In England, while breastfeeding initiation rates have steadily increased in recent years from 76% in 2005 to over 80%, early breastfeeding cessation is still prevalent with only an estimated 47.6% of infants ages 6–8 weeks old being breastfed (including combination feeding with formula) in 2020/2021 [1, 2]. Early breastfeeding cessation in high-income settings is associated with various factors, such as negative breastfeeding experiences [3, 4], difficulties feeding [5, 6], limited access to breastfeeding resources [7], and experience of social pressure [8]. When provided consistently and adequately, social support has been shown to mitigate these factors [9], resulting in increased breastfeeding durations [10] with benefits for infants’ long-term health and wellbeing [11]. Maternal mental health, which is closely intertwined with infant feeding experiences [8, 12], is also important. When infant feeding experiences do not meet with expectations, mothers may experience emotional distress and be at increased risk of postnatal depression [12, 13]. It is in the public health interest to help individuals reach their breast goals. To achieve this, UK governments have noted the importance of effective social support for breastfeeding by health care professionals, as outlined within key guidance such as England’s Healthy Child Programme and Scotland’s Child Health Programme [14–16].

Social support is characterized by either the *experience* or *perception* of the sharing of resources from one group or individual by a recipient [17]. This may consist of practical, informational, appraisal or emotional assistance, and come from family, friends, professionals, or the wider community. A recently updated Cochrane review of breastfeeding interventions across the globe found that support from health care professionals was associated with increased breastfeeding duration, particularly when the support was face-to-face [18]. In the UK, health visitors are identified as key providers of informational and emotional support to parents, to address low breastfeeding rates at the local level [14, 19, 20]. Health visitors are registered nurses or midwives who have received additional training in community public health to provide care for all families in the community. For example, in England, health visitors work with families during five mandated visits from pregnancy to five years postpartum, with these visits typically running alongside additional community services (such as weight and child health clinics) and enhanced support targeting potentially vulnerable children, women, and families. Their mandated first visit usually takes place in the home (prior to COVID-19 related guidance) within 14 days of birth, and the second visit is expected to occur at around 6 to 8 weeks [21]. This face-to-face, in-home interaction during the early postpartum period when many women stop exclusive breastfeeding [13] makes health visitors key practitioners to provide support to establish and continue breastfeeding [22]. Health visitor support can take the form of informational support, where they provide information and guidance on feeding, providing advice to address common problems, and refer parents to more resources if needed [22, 23]. Health visitors may also be an important source of emotional support, most easily understood as empathy and connectedness [17], which is associated with increased parental confidence, self-efficacy, and perception of future support availability [9, 24–27].

Despite UK health policies placing support and health visitors at the centre of the strategy to improve breastfeeding rates, breastfeeding rates remain stubbornly low, highlighting room for improvement [11]. In terms of health visitors, there may be structural issues – funding challenges, increased caseloads, and inadequate resources – limiting their capacity to provide adequate support [28]. A 2015 report found that 25% of families in England were not receiving their mandatory visits, and in some regions health visitors reported a 16% increase in their caseloads, far exceeding recommendations [29]. In addition, a key issue may be an over-focus on information delivery as a form of support: UK-focused systematic reviews have found that provision of support, primarily informational in nature, has had limited impact on breastfeeding rates beyond initiation [10, 30, 31]. While many mothers actively request advice about how to breastfeed [18, 32], advice about breastfeeding can be morally loaded [33–37] and so may not be experienced as supportive. By focusing on the promotion of the “…health benefits of breastfeeding as well as the risks of not breastfeeding” [15], mothers may feel both pressured to breast/chest-feed and a lack of empathy, particularly if they are not wishing to or struggling to do so. A recent prospective observational study of first-time mothers in Italy found that 80% of participants experienced difficulty breastfeeding when they received greater informational than emotional support [38]. Emotional support may reduce stress, which may improve breastfeeding outcomes via psychobiological pathways including lowering maternal cortisol levels leading to greater breastmilk supply/energy density [39, 40].

Overall, having emotional support present in conjunction with informational support is likely important in the positive reception and application of advice. Thus, here in a sample of 565 UK self-identified mothers, we test the hypothesis that emotional support moderates the relationship between informational support and infant feeding outcomes. We predict that 1) informational and emotional support are associated with a) a decreased hazard of breastfeeding cessation prior to 3 months and b) a positive infant feeding experience; 2) the effect of informational support on both outcomes will be stronger when mothers receive emotional support.

## Methods

This research project was approved by the LSHTM Ethics Committee (reference number 25649). The study from which the data stems, the “Social Support and Feeding your Baby” survey, obtained ethics approval from the UCL Research Ethics Committee in 2017. In the initial questionnaire informed consent was obtained regarding the future use of anonymized data for projects that fit the original scope.

### Data

A retrospective online survey collected data between December 2017 – February 2018. Convenience sampling was used to recruit participants self-identifying as mothers who had given birth (in the last 24 months in the UK) through social media sites and parenting forum websites. This sampling method could present issues with generalizability that are addressed in the limitations. While the survey itself used breastfeeding-related language, we did not collect data regarding the gender identification of participants and acknowledge that this may not have been our participants’ preferred descriptor. Only responses from participants who reported the variables of interest were used: from the initial survey of 738 eligible participants 625 mothers responded to questions concerning duration; however, 45 did not answer breastfeeding initiation questions and 14 did not respond to social support questions, resulting in a final sample of 565 for the analysis of breastfeeding duration [5], while a further 7 did not answer the experience questions (*n* = 558). We retain 3 women in the sample whose infant was in their 25th month at time of the survey. An overview of the definitions for each variable used, including how they were measured in the initial survey, can be seen in Table 1*.*

**Table 1:** Overview of variables and the survey questions they originate from, adapted from [4]

Variable	Survey question
*Outcome – Infant feeding related measures*	
Duration of any breastfeeding	Did you ever breastfeed your youngest child(ren) (including expressing)? *Yes, No, Prefer not to say* Are you currently providing any breastmilk to your youngest child(ren), either exclusively or alongside formula and/or solids? *Yes, No* Approximately, how long did you provide any breastmilk your youngest child(ren)? *Specify number and select unit (days, weeks, months)*
Maternal subjective experience	How would you describe your overall experience around feeding your youngest child(ren)? Please tick all that apply. *Option list included: ‘enjoyable’ and ‘rewarding’, ‘good for bonding’, ‘hard work’, ‘stressful’, ‘emotionally draining’, ‘none of the above’, and ‘prefer not to say’*
*Exposure – Health visitor social support measures*	
Informational support	Thinking back to the first few weeks after giving birth to your youngest child(ren)… on a scale of 1 to 5 with 1 being "very helpful" and 5 being "very unhelpful," how helpful did you find the health visitors advice and/or information overall? *‘very helpful’, ‘helpful’, ‘neither helpful nor unhelpful’, ‘unhelpful’, ‘very unhelpful’, ‘not applicable’*
Emotional support	Thinking back to the first few weeks after giving birth to your youngest child(ren)…how emotionally supported did you feel by your health visitor? ‘*very supported’*, ‘*supported’*, ‘*neither supported nor unsupported’*, ‘*unsupported’*, ‘*very unsupported’*, ‘*not applicable’*
*Controls*	
Age of mother	In what year were you born (yyyy)
Educational attainment	What is your highest qualification level? *GCSEs or equivalent, AS/A-levels or equivalent, Graduate or equivalent, Postgraduate or equivalent, Other*
Intention to breastfeed	I planned to breastfeed my baby(ies). *Yes, no*
Breastfeeding exposure	Thinking back to before you gave birth to your youngest child(ren), what were your thoughts and experiences around feeding your baby(ies)? Please select all that apply… ‘*I knew people who were breastfeeding / had breastfed their baby(ies)’*
Mother breastfed	Were you breastfed as a baby? ‘*Yes’, ‘no’, ‘don’t know’*
Parity	In total, how many children do you have?
Annual income	Approximately, what is your total annual household income? ‘*Less than £10,000’, ‘Between £10,000 and £20,000’,’ Between £20,000 and £30,000’, ‘Between £30,000 and £40,000’, ‘Between £40,000 and £50,000’,’ Between £50,000 and £60,000’, ‘Between £60,000 and £70,000’, ‘Between £70,000 and £80,000’, ‘£80,000 or more’, ‘don’t know’, ‘Prefer not to say’*

#### Health visitor support

The exposure variables, informational and emotional support, measure the supportiveness of health visitors during the early postnatal period. The informational support response options were collapsed into a binary variable where the options ‘not applicable’, ‘neither helpful or unhelpful’, and ‘unhelpful’ and ‘very unhelpful’ were combined into a new ‘*not helpful*’ category, while ‘helpful’ and ‘very helpful’ were combined into a new ‘*helpful*’ category. Emotional support was similarly collapsed, with ‘not applicable’, ‘neither supported or unsupported’, ‘unsupported’, and ‘very unsupported’ combined into a new ‘*not supported*’ category, and ‘supported’ and ‘very supported’ combined into ‘*supported*’. Exploratory analysis of descriptive statistics (see Figure S1 and Table S1-2) indicates that the grouping of negative and neutral response categories is justifiable in relation to our outcome variables, as they are quantitatively similar to each other and dissimilar to the positive response categories.

#### Duration of any breastfeeding outcome variable

If participants reported having ever breastfed their youngest child, they were asked if they were still giving any breastmilk (either exclusively, supplemented with formula or solid foods) and if they had stopped, how long they breastfed for (in days, weeks, or months).

#### Experience outcome variables

Maternal subjective experience of infant feeding was assessed using six response options measuring different dimensions, which can be broadly split into either *positive* – ‘enjoyable’, ‘rewarding’, ‘good for bonding’ – or *negative* – ‘stressful’, ‘hard work’, ‘emotionally draining’. While these dimensions can broadly be broken into positive or negative experiences, none are mutually incompatible (e.g., a participant may report finding infant feeding both stressful and good for bonding [4]). Each dimension is scored separately as either 0 = not reported or 1 = reported.

#### Control variables

Our final sample was relatively highly educated compared to the general population, with 81.04% having received a graduate or postgraduate qualification. Given this distribution we reduced the levels in this variable to two; participants were recorded as having a higher education (coded as 1) or not (coded as 0). For the variable annual income 35% reported incomes above £60,000 per year. Prior to analysis this variable was reduced to a binary variable “high earner”, coded as 0 if annual income was below £50,000 or 1 if above it, reflecting the median point in our data (46.2% reported incomes below or at £50,000-£60,000). Most participants reported having one (61.2%) or two children (32.6%). For analysis this was transformed into a binary parity variable (0 = one child, 1 = two or more children). Most participants planned to breastfeed (93.6%) (breastfeeding intention coded as 0 = did not plan to breastfeed, 1 = planned to breastfeed). Likewise, prior breastfeeding exposure was reported by the majority (80.57%) (0 = no exposure, 1 = known others to breastfed). Finally, whether the participant themself was breastfed was coded as 1 = yes (70.5%), 2 = no (4.1%), or 3 = don’t know (25.4%). Note, these descriptive statistics are based on the larger duration analysis sample (*n* = 565).

### Statistical analysis

All the statistical analyses were conducted in R (version 4.0.5) [41] using the packages *survival* [42], *survminer* [43] for the cox regressions and base R for the generalized linear models. The dataset supporting the conclusions of this article is available in the OSF repository, https://osf.io/2d4v5/.

#### DAGs (Directed Acyclic Graphs)

We take a directed acyclic graph (DAG) approach to model selection [44] using the R package dagitty [45], with separate DAGs for the duration and experience analysis used to identify the minimally sufficient adjustment set of variables to control for. Other variables included in the starting DAG included (Table 1): breastfeeding intention, annual income; educational attainment, mother breastfed, parity, breastfeeding exposure, and age of mother. From our starting DAGs, we tested the implied conditional independencies among variables (Johannes et al., 2016) using the lavaan package [44, 46] and updated the graphs where unanticipated statistically significant relationships existed within our sample that could reasonably be inferred to be the result of direct causality (see SI code). After updating, the minimally sufficient adjustment set from each graph was calculated, identifying in the following control variables: 1) *duration analysis* – breastfeeding intention, educational attainment, and parity; *2) experience analysis* – age of mother, parity, and mother breastfed (see Figure S2)*.*

#### Duration analysis

To test our predictions regarding breastfeeding duration we use a Cox regression model, assessing the hazard of breastfeeding cessation before three months (13 weeks) dependent on informational and emotional support from health visitors (*n* = 565). Participants who reported breastfeeding for longer than three months or were still breastfeeding and the child was older than three months were right censored. The reported model includes main effects for informational and emotional support and an interaction term between the two. The non-proportional hazards assumption was confirmed for all models.

#### Experience analysis

Binary logistic regression models were used to test our predictions regarding experience. The six experience outcomes variables were tested in separate models, each of which included main effects for informational and emotional support and an interaction term between the two types of support (*n* = 558).

## Results

### Descriptive statistics

On average, participants (*n* = 565) were aged 32.28 (SD = 4.37) years and they had a mean number of 1.46 (SD = 0.64) children. The mean age in weeks of the focal child was 50.29 (SD = 27.73), the youngest being 1.86 weeks and the oldest 106.29 weeks old. At the time of the survey, 223 participants (41.16%) were still providing breastmilk, and 450 (79.51%) had continued breastfeeding for up to three months. Overall, the majority reported infant feeding to be ‘hard work’ (70.25%), ‘good for bonding’ (73.84%), ‘rewarding’ (69.0%) and ‘enjoyable’ (65.23%). Fewer participants, but still a notable proportion reported infant feeding to be stressful (46.95%) and emotionally draining (44.44%). Most participants (58.76%) reported emotional support from health visitors in the first weeks following childbirth to be ‘very supportive’ (22.48%) or ‘supportive’ (36.28%), while 14.16% women reported this support to be ‘neither supportive or unsupportive’ and 10.97% reported it to be ‘unsupportive or very unsupportive’. Similarly, 24.96% of participants reported the information and advice provided by health visitors to be ‘very helpful’ and 36.46% said it was ‘helpful’, while 14.51% reported it to be ‘neither unhelpful or helpful’ and 10.44% reported it to be ‘unhelpful’ or ‘very unhelpful.’

### Duration analysis

**Fig. 1:**
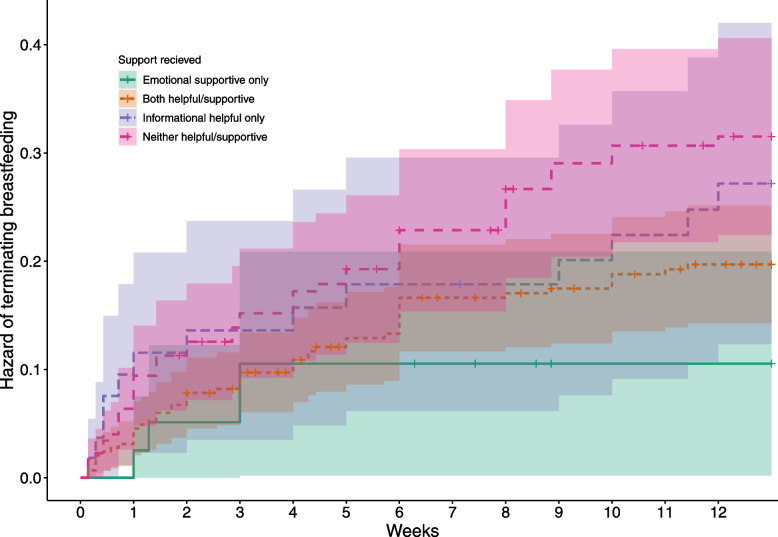
Hazard of terminating breastfeeding from 0 – 13 weeks by support type (*n* = 565). Crosses indicate right censored events. The solid green line represents when both emotional support was supportive, but informational support reported as unhelpful. The small, dashed orange line represents when both information and emotional support were reported to be helpful/supportive. The dashed purple line is when informational support was reported as unhelpful and emotional support as unsupportive. The pink dashed line with larger gaps is when neither informational and emotional support were reported to be helpful/supportive

The highest hazard of breastfeeding cessation was associated with *both* informational and emotional support being reported either negatively or absent (HR = 2.84) (Fig. 1). However, unexpectedly, this did not differ to when both categories of support were reported positively (HR = 2.408, *p* = 0.154, 95% CI [0.720, 8.048]). Instead, the lowest hazard of breastfeeding cessation prior to 13 weeks was associated with emotional support categorised as ‘supportive’ and informational support as ‘not helpful’: the hazard of cessation in any given week was 64.8% lower in this scenario compared to when both were reported negatively or as absent (HR = 0.352, *p* = 0.047, 95% CI [0.126, 0.984]). Helpful informational support combined with unsupportive or absent emotional support was not found to differ statistically from when both were reported negatively or as absent (HR = 0.711, *p* = 0.287, 95% CI [0.379, 1.333]). Full result tables can be found in the SI.

### Experience analysis

**Fig. 2:**
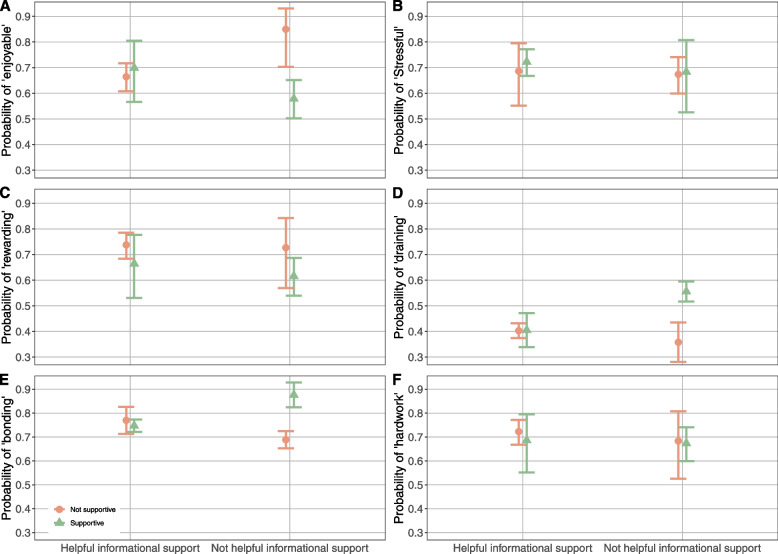
Predicted probability from the six logistic regression models for the following infant feeding experiences: **A**) Enjoyable, **B**) Stressful, **C**) Rewarding, **D**) Emotionally draining, **E**) Good for bonding and **F**) Hard work. Informational support is on the x-axis and the probability of reporting (0–1) the modelled experiences on the y-axis. Triangle (green) point estimates and error bars (95% CI) represent ‘supportive’ emotional support; circle (orange) points are when emotional support was reported as ‘not supportive’. *N* = 558

For all models except ‘hard work’, the point estimate for the predicted probability of positive infant feeding experience was *lowest* (Fig. 2A, C, E) and for negative experience was *highest* (Fig. 2B, D) when ‘not helpful’ informational support was combined with ‘not supportive’ emotional support.

Across models, health visitors being emotionally supportive did not appear to be associated with additional benefits when they were also considered informationally helpful; point estimates for the predicted probability of a given experience were similar and, except for the ‘rewarding’ model, 95% CIs for emotionally ‘supportive’ fell entirely within those of emotionally ‘not supportive’. However, when health visitors were reported to be *not* informationally helpful, their being nonetheless emotionally supportive was associated with similar or *better* outcomes to when they were rated positively on both counts. Better subjective experience of infant feeding in association with health visitors being considered emotionally supportive, when their informational support was deemed not helpful, was found most compellingly in relation to the following dimensions: ‘Enjoyable’ – OR = 2.854, *p* = 0.020, 95% CI [1.236, 7.75] compared to both reported positively and OR = 4.115, *p* = 0.003, 95% CI [1.743, 11.383] compared to both reported negatively or absent; ‘Good for bonding’ – OR = 3.205, *p* = 0.022, 95% CI [1.283, 9.772] compared to both reported negatively or absent; and ‘Emotionally draining’ – OR = 0.445, *p* = 0.028, 95% CI [0.211, 0.906] compared to both reported negatively or absent.

## Discussion

These results highlight the importance of health visitors providing emotional support, in addition to informational support, in the early postnatal period to bolster the continuation of breastfeeding and encourage a positive subjective experience of infant feeding. Previous studies from the UK have found that social support, typically consisting of informational support, has not been particularly effective at improving breastfeeding duration [10, 30, 31]. While we did not find emotional support to moderate the effect of informational support, these findings suggest emotional support may be crucial in improving breastfeeding duration/experience, adding to an emerging consensus that professional support should involve more than just the delivery of information to ensure optimal mother-infant outcomes.

As expected, the hazard of breastfeeding cessation was consistently highest over time when participants reported that health visitor support was both informationally and emotionally not helpful. Typically, research to date has focused on the importance of informational support from health visitors for increasing breastfeeding durations [22, 38, 47, 48]. However, informational support from health visitors is not always considered helpful by recipients for a range of reasons [10, 30, 31] and these results suggest that experiencing health care professionals as emotionally supportive despite this can improve breastfeeding durations. Emotional support, in the form of providing encouragement, validation of difficult circumstances, and judgement-free support of a mother’s infant feeding practices can go a long way [22, 38]. In particular, in high-income settings like the UK where mothers often rely on expert parenting advice, receiving emotional support from health care professionals can be an important facilitator of breastfeeding education enactment and empowerment [49].

While at first glance it may be surprising that participants who reported their health visitors to be emotionally supportive but informationally unhelpful had *longer* lactation durations than those who reported both informational and emotional supported positively, this may reflect a lack of need for informational support among these participants [5, 50]. Earlier work with this sample has found that support clusters [48], suggesting participants with supportive health visitors were also likely to have ready access to other supporters who may duplicate information, dampening the perceived utility of their advice [51, 52]. Affluent mothers, who predominate in our sample, typically have privileged access to support and information, and are likely to breastfeed for longer [53, 54]. Informational support may also only be considered helpful if it solves a specific problem; parents without problems requiring assistance may give lower helpfulness ratings due to the nature of their experience, rather than the content or provision of support received. Since breastfeeding problems predict cessation [5], it may be that participants reporting health visitors as ‘emotionally supportive *and* informationally not helpful’ experienced fewer problems. Conversely, when problems are encountered not all information considered helpful by parents is likely to encourage persistence with breastfeeding and may in fact have the opposite effect [5]. These explanations are not mutually exclusive, nor do they diminish the importance that should be placed on emotional support.

In terms of the subjective experience of infant feeding, not all problems, breast/chest-feeding-related or otherwise, can be adequately addressed or surmounted with information; when this is the case, the determining factor is likely to be emotional support. This is bolstered by the finding that when health visitors were reported to be not informationally helpful, their being nonetheless emotionally supportive was associated with similar or *better* outcomes compared to when they were rated positively on both counts. Experiencing emotional support appears to be particularly associated with finding infant feeding enjoyable, good for bonding, and not emotionally draining, with implications for both parental emotional wellbeing and infant outcomes. Though our measure of infant feeding experience is not specific to breastfeeding, relatively high rates of prolonged breastfeeding in this sample suggest emotional support is likely to enhance the experience of both it and other modes of infant feeding.

### Recommendations for best practice for health visitors

Looking at how health visitors provide support can help avoid the poorer outcomes for infant feeding, so it is essential to focus on which aspects of informational and emotional support health visitors can best provide to women. For best practice in terms of informational support, health visitors should be supported to provide the most up-to-date, non-judgmental, non-conflicting and relevant information for the individual and their desired infant feeding methods. Previous research has highlighted that morally loaded, contradictory information can increase the challenges and frustrations associated with infant feeding, undermining women’s ability to continue breastfeeding, feel confident formula feeding, and harm mental health [12, 33, 35–37, 55–58]. Qualitative reports from our participants highlight this, for example in response to the open text question asking if there was anything about their postnatal experiences they would like to share, one mother responded: "*My health visitor was useless. In fact, at times, she made me feel inadequate and gave me incorrect/out-of-date information*." Ensuring that health visitors are able to offer effective strategies to navigate challenges and difficulties from the initiation until the cessation of breastfeeding can help facilitate a positive breastfeeding experience [24, 52], while those who are formula feeding must also not be forgotten [4]. It is also important for health visitors to quickly and actively refer women to specialist breastfeeding councilors such as lactation consultants -provided they are available- to ensure mothers have access to high quality informational support.

Our results also suggest it is also key that emotional support is provided alongside the informational support, which has been the mainstay of health visitor support [22]. Health visitors should be supported to provide reassurance to mothers, and employ tactics such as encouragement, validation of difficult circumstances and judgement-free support of a mother's infant feeding choices. It is important to support the mother's mental health to ensure that not only do women feel like they can breastfeed, but they also have the emotional support to feed their infants how they wish while ensuring their mental well-being [5]. This suggestion likely entails structural changes to the provisioning of health visiting services in the UK. For example, in England, a recent report highlights the loss of funding and cuts to the number of health visitors since 2015 when responsibilities were transferred to local authorities [59]. New staff are not being trained replace these losses, increasing the caseloads of existing health visitors, particularly in dense urban areas like London [60]. As a result, health visitors who continue are overworked, under pressure, experience low morale and thus have less capacity to offer quality emotional support [60]. These known pressures have been amplified due to the COVID-19 pandemic, further limiting Health Visitors’ ability and capacity to provide support [61]. In addition, these structural pressures limit health visitors’ ability to engage in further training and research about how best to support mothers, hindering the transmission of best practice.

### Limitations

One of the primary limitations of this sample is its homogeneity, consisting of primarily white and highly educated women in the UK. This can be attributed to the use of convenience sampling, and our data are not representative of the current breastfeeding population in the UK. This means our findings cannot be generalised across the UK and beyond, especially to those with lower socioeconomic positions (e.g., lower education) who may experience different pressures around breastfeeding as evidenced by findings that they disproportionately experience lack of support during breastfeeding [62]. This limitation is important to keep in mind for future studies on health visitor support, with the need to prioritise diverse samples of participants to ensure results reflect the socio-economic and cultural diversity within the UK. Further, our survey data are retrospective in nature, risking recall bias. For example, studies show mothers are more likely to overestimate their breastfeeding duration with retrospective reports [63]. High quality prospective data collection on infant feeding and support is currently lacking, and it is critical that such studies are supported to better understand the pathways to better breastfeeding outcomes in the UK.

## Conclusion

These results indicate the importance of health visitors’ emotional support in both the maintenance of breastfeeding and the subjective experience of infant feeding more generally. They highlight an area in which health visitors can improve the high rates of early breast/chest-feeding cessation within the UK. Informational support will always be a crucial component of a health visitor's job; however, emotionally supporting parents through the physically and mentally exhausting postnatal period is also key to improving both feeding and mental health outcomes. A more holistic approach to "support" is needed, requiring an overhaul of health visitors’ current practice. Introducing new policy that provides training in emotional counselling could be effective, along with the allocation of more funding for health visitors. Health visitors are vital members of breast/chest-feeding support; however, they too require support, in the form of an increased labour force and funding, to overcome current barriers to providing adequate care to all new parents in the UK [28].

## Supplementary Information

**Additional file 1.**

**Additional file 2.**

## Data Availability

The dataset supporting the conclusions of this article is available in the OSF repository, https://osf.io/2d4v5/.
