# Supplementary material for: Emotional and informational social support from health visitors and breastfeeding outcomes in the UK
Source: Int Breastfeed J. 2023 Mar 7;18:14. doi: 10.1186/s13006-023-00551-7 (PMC9990566; doi:10.1186/s13006-023-00551-7)
Supplement: Supplementary file 1 — Additional file 1. [file 13006_2023_551_MOESM1_ESM.docx]

**SI:** **Measuring the contribution of emotional and informational social support from health visitors to breastfeeding duration and infant feeding experience in the UK**

**Authors: Chambers, A^1^, Emmott, E. H.^2^ and Myers, S.^2,3^**, **Page, A. E.^1^,**

**Affiliations:**

^1^ London School of Hygiene and Tropical Medicine

^2^ UCL Anthropology, University College London, 14 Taviton Street, London, WC1H 0BW

^3^ BirthRites Independent Max Planck Research Group, Max Planck Institute for Evolutionary Anthropology, Leipzig

**Keywords:** infant feeding; social support; breast/chest-feeding; health visitors; UK

**Figures**


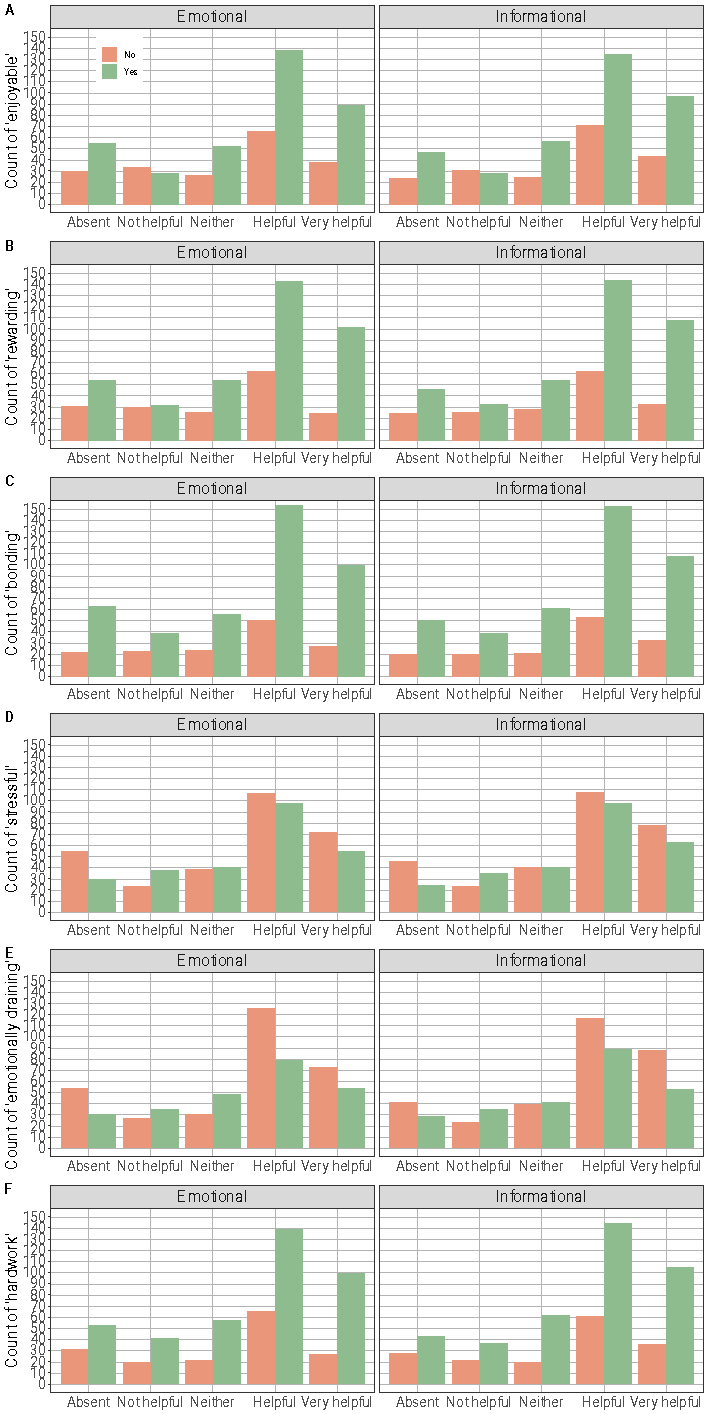


Figure S1: frequency of reported outcomes for emotional and information support across the 6 subjective experience outcomes. Orange bars (left) represent the negative response (i.e., when participants did not record that infant feeding was ‘rewarding’). The green bars (right) represent the positive response (i.e., when participants recorded infant feeding as ‘hard work’).


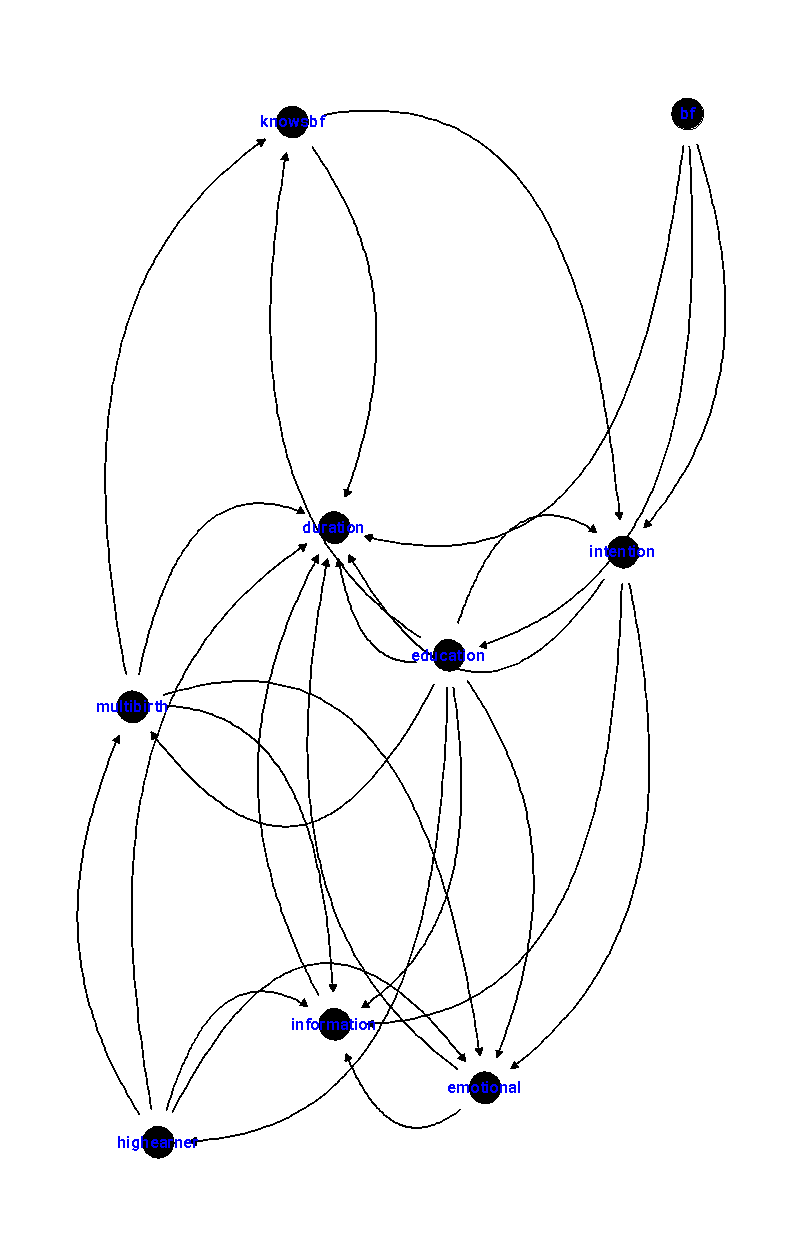


Figure S2: final DAG used to model breastfeeding duration. Variable names represent: emotional = emotional support, information = informational support, highearner = annual income, multibirth = number of children, education = educational attainment, duration = duration of breastfeeding, intention = planned to breastfeed, knownsbf = if knows people who breastfeed and bf = if breastfed themselves as an infant.

|  | Breastfeeding cessation before 13 weeks (Column %) | Continue to breastfeed for 13 weeks  (Column %) |
| --- | --- | --- |
| **Emotional Support** |  |  |
| Absent | 70 (15.6) | 22 (19.0) |
| Supportive or very supportive | 277 (61.6) | 55 (47.4) |
| Neither supportive or unsupportive | 60 (13.3) | 20 (17.2) |
| Unsupportive or very unsupportive | 43 (9.6) | 19 (16.4) |
| Total | 450 | 116 |

Table S1: Descriptive tables for emotional support cross tabulated with breastfeeding duration separated into less than 13 weeks and greater than 13 weeks. Cell Count and (column %) is in brackets.

|  | Breastfeeding cessation before 13 weeks (Column %) | Continue to breastfeed for 13 weeks  (Column %) |
| --- | --- | --- |
| **Informational Support** |  | |
| Absent | 60 (13.3) | 18 (15.5) |
| Supportive or very supportive | 283 (62.9) | 64 (55.2) |
| Neither supportive or unsupportive | 62 (13.8) | 20 (17.2) |
| Unsupportive or very unsupportive | 45 (10.0) | 14 (12.1) |
| Total | 450 | 116 |

Table S2: Descriptive tables for emotional support cross tabulated with breastfeeding duration separated into less than 13 weeks and greater than 13 weeks. Cell Count and (column %) is in brackets.
